# Amino Acid Polymorphisms on the Brazilian Strain of Yellow Fever Virus Methyltransferase Are Related to the Host’s Immune Evasion Mediated by Type I Interferon

**DOI:** 10.3390/v15010191

**Published:** 2023-01-10

**Authors:** Nathália Dias Furtado, Iasmim Silva de Mello, Andre Schutzer de Godoy, Gabriela Dias Noske, Glaucius Oliva, Bruno Canard, Etienne Decroly, Myrna C. Bonaldo

**Affiliations:** 1 Laboratório de Biologia Molecular de Flavivírus, Instituto Oswaldo Cruz-Fiocruz, Rio de Janeiro 21040-900, Brazil; 2 Centro de Pesquisa e Inovação em Biodiversidade e Fármacos, Instituto de Física de São Carlos-USP, São Paulo 13563-120, Brazil; 3 Architecture et Fonction des Macromolécules Biologiques, Aix-Marseille Université, CNRS, UMR7257, 13009 Marseille, France

**Keywords:** yellow fever virus outbreak, methyltransferase, virulence, type I interferon

## Abstract

Since late 2016, a yellow fever virus (YFV) variant carrying a set of nine amino acid variations has circulated in South America. Three of them were mapped on the methyltransferase (MTase) domain of viral NS5 protein. To assess whether these changes affected viral infectivity, we synthesized YFV carrying the MTase of circulating lineage as well as its isoform with the residues of the previous strains (NS5 K101R, NS5 V138I, and NS5 G173S). We observed a slight difference in viral growth properties and plaque phenotype between the two synthetic YFVs. However, the MTase polymorphisms associated with the Brazilian strain of YFV (2016–2019) confer more susceptibility to the IFN-I. In addition, in vitro MTase assay revealed that the interaction between the YFV MTase and the methyl donor molecule (SAM) is altered in the Brazilian MTase variant. Altogether, the results reported here describe that the MTase carrying the molecular signature of the Brazilian YFV circulating since 2016 might display a slight decrease in its catalytic activity but virtually no effect on viral fitness in the parameters comprised in this study. The most marked influence of these residues stands in the immune escape against the antiviral response mediated by IFN-I.

## 1. Introduction

Yellow fever virus (YFV) is the causative agent of an acute febrile disease endemic to Sub-Saharan Africa and South America. Since the beginning of the 21st century, small events of YFV spreading were described in Brazil, which resulted in a significant and unprecedented outbreak in late 2016, peaking between 2017 and 2018 [1,2,3]. Phylogenetic analyses described the clustering of all the YFV sequences determined since 2004 in a sub-clade 1E of the modern lineage belonging to the South American I clade [4,5,6]. During the latest Brazilian outbreak, complete sequenced genomes of YFV from samples of naturally infected mosquitos, non-human primates, and humans displayed a molecular signature consisting of polymorphisms associated with the mutation of nine amino acids [2,6]. Three amino acid markers are located in the methyltransferase (MTase) domain of viral protein NS5 at positions R101K, I138V, and S173G [6].

In eukaryotes, mRNA capping and methylation is a pivotal post-transcriptional modification, promoting the mRNA translocation from the nucleus to the cytoplasm and limiting RNA degradation [7]. These post-transcriptional modifications are essential for viral RNA translation, replication, and stability. YFV belongs to the genus *Flavivirus* and family Flaviviridae, displaying a positive single-stranded RNA composed of a unique open reading frame flanked by two untranslated regions (5′- and 3′-UTR) [8]. The replication cycle of flaviviruses occurs in the host’s cell endoplasmic reticulum-derived membrane without an entry into the nucleus [9]. Consequently, these viruses carry their RNA capping machinery, which involves the N-terminal MTase domain of the NS5 protein [10,11].

The flavivirus NS5 MTase is a globular domain that catalyzes the cap formation with a still ill-defined guanylyltransferase (GTase) activity and its subsequent methylation by two methyltransferase activities; the guanine-N7-methylation and the 2′-O-nucleoside methylation [11,12,13]. It is believed that the GTase activity transfers a GMP moiety of a GTP molecule to the 5′-diphosphate viral RNA (ppAGN), generating pyrophosphate (PPi) as a by-product, and the capped RNA, GpppAGN. The ppAGN results from the hydrolysis of pppAGN to ppAGN releasing a phosphate (Pi) by the RNA triphosphatase activity of the NS3 protein [14]. After viral RNA capping completion, the MTase catalyzes two sequential methylation reactions [13]. First, MTase transfers a methyl group from the methyl donor S-adenosyl-L-methionine (SAM) to the N7 position of the guanosine cap. This step generates a cap-0 structure [15] (m7GpppAGN) and the S-adenosyl-L-homocysteine (SAH) by-product. The MTase binds next to another SAM molecule and transfers its methyl group to the 2′-hydroxyl position on the ribose of the first viral genomic RNA nucleotide, the conserved adenine (A) nucleotide, to form the cap-1 structure [7] (m7GpppAmGN) [13,16,17].

Crystallographic studies on NS5 MTase domain of flaviviruses such as dengue virus [11], West Nile virus [16], Meaban virus [18], Japanese Encephalitis virus [17], Zika virus [19], Wesselsbron virus [20], Murray Valley Encephalitis virus [21] and YFV [22] have allowed locating the binding sites for GTP, SAM/SAH, and viral RNA. Only one binding site for the methyl donor, SAM, was identified in these structures, where the two methylation reactions occur [13]. The mechanism proposed for completing both MTase activities involves repositioning the nascent viral RNA and substituting the SAH molecule for a SAM molecule after the N7-methylation, thus engaging conformational rearrangement [16,23].

Mutational screening studies indicated that mutations impairing the 2′O-methylation of viral RNA attenuated West Nile virus replication upon cell transfection, whereas mutations blocking the N7-methylation abolished viral replication [24]. Considering that RNA methylation is essential to viable viral infection and the differences between host cell and flaviviral cap formation processes, it is possible to lead antiviral research by targeting this domain of the viral protein NS5 [16]. Different strategies for MTase inhibition have been developed. A targeted region of the MTase is the SAM binding site. Several SAM analogs, such as Sinefungin, have been designed and used to inhibit the MTase activity [16,25,26,27].

In addition to its role in capping, which is central for viral protein expression and RNA protection against degradation 5′ exonucleases, the MTase also plays a role in the host’s innate immune response evasion [28,29]. Indeed, it has been recently demonstrated that the 2′O-methylation of the cap structure is a self-marker [30,31]. Thus, RNA lacking N1 2′O methylation is detected by RIG-I, leading to the production of type 1 interferons (IFN-1). Furthermore, this mis-capped RNA is sequestered by IFIT proteins [32,33], thereby limiting viral replication. The MTase domain of the NS5 protein has been shown to interact directly with proteins involved in the interferon pathway, such as STAT2, which is involved in the interferon-alpha/beta receptor (IFNAR) signaling and allows the escape from the antiviral response [32,34,35,36].

The relevance of genetic modifications in positive single-stranded RNA viruses has been widely explored using reverse genetic methods [37,38]. Here, we synthesized YFV based on the genomic sequence of a YFV isolated in Brazil in 2017. We conducted site-directed mutagenesis to revert the molecular signature residues located in the MTase domain of NS5, generating two YFV variants. Using this approach, we addressed the effects of amino acid polymorphisms in viral fitness in vitro and in vivo. Next, we investigated the enzymatic activity of two isoforms of YFV NS5 bearing at positions 101, 138, and 173, the residues R, V and G, or K, I and S, respectively, through protein purification. Our results suggest that the mutations of the Brazilian strain might play a role in binding the methyl donor SAM to the MTase and is related to higher sensitivity to IFN-I.

## 2. Materials and Methods

### 2.1. Comparative Modeling of YF MTase 2017

**Figure 1:**
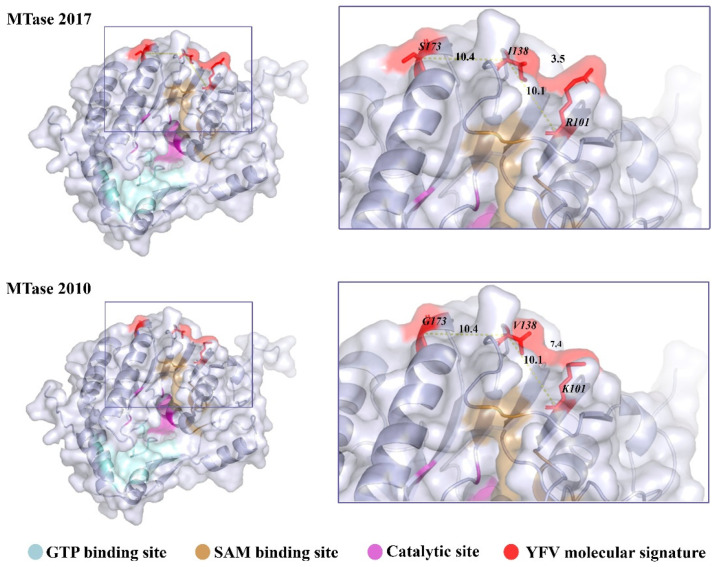
Localization of the YFV 2016−2019 molecular signature in the NS5 MTase domain. The MTase domain of the NS5 was modeled by homology based on the sequence of YFV isolate ES-504 (GenBank: KY885000) and on the crystallographic data of YFV 17D MTase (PDB: 3EVB), MTase 2017. Mutations were inserted in PyMOL, generating MTase 2010. The models are represented in the cartoon with their respective molecular surfaces. GTP (blue) and SAM (pale yellow) binding sites are highlighted, as well as the catalytic site (purple) responsible for the methyl transfer reactions and the residues that compose the YFV 2016−2019 molecular signature.

We modeled the NS5 MTase domain of the prototype YFV isolated in 2017 (GenBank: KY885000) and the variant carrying the three amino acid changes (R 101 K, V 138 I, and G 173 S) using Modeller software version 10.1. The search for a suitable template was performed on the Swiss-model server [39]. The PDB structure from the vaccine YFV complexed with S-adenosyl-L-homocysteine (SAH; 3EVB) [22] was employed as a template, sharing 94.32% identity with the YFV 2017 sequence. The template and target sequences were aligned using the "align2d” function of Modeller [40]. A total of 100 models were obtained for each MTase variant using the “automodel” routine of the Modeller. Each model was optimized using the variable target function method (VTFM) for 300 iterations and molecular dynamics (MD) in slow mode. The entire cycle was repeated twice to ensure the optimized conformation of the generated model. The resulting modeled structures were ranked according to their discrete optimized protein energy (DOPE) score. PROCHECK further assessed the best model (the lowest DOPE score; Figure 1) in SAVES v.6 servers for the Ramachandran plot and ProSA to verify the model’s overall quality through z-score [41,42]. The PyMOL Molecular Graphics System, Version 2.0 Schrödinger, LLC, was used for structure visualization.

### 2.2. Synthesis of YFV cDNA Infectious Clone

The methodology employed in this study was based on the strategy developed for the infectious clone Zika virus Rio-U1 [43]. The cDNA genome was derived from the YFV isolate ES-504 (GenBank: KY885000). This YFV was isolated from a non-human primate belonging to the *Alouatta guariba clamitans* species in the state of Espírito Santo, Southeastern Brazil, in February 2017. The cDNA was previously obtained by RT-PCR and sequenced [2,6].

**Figure 2:**
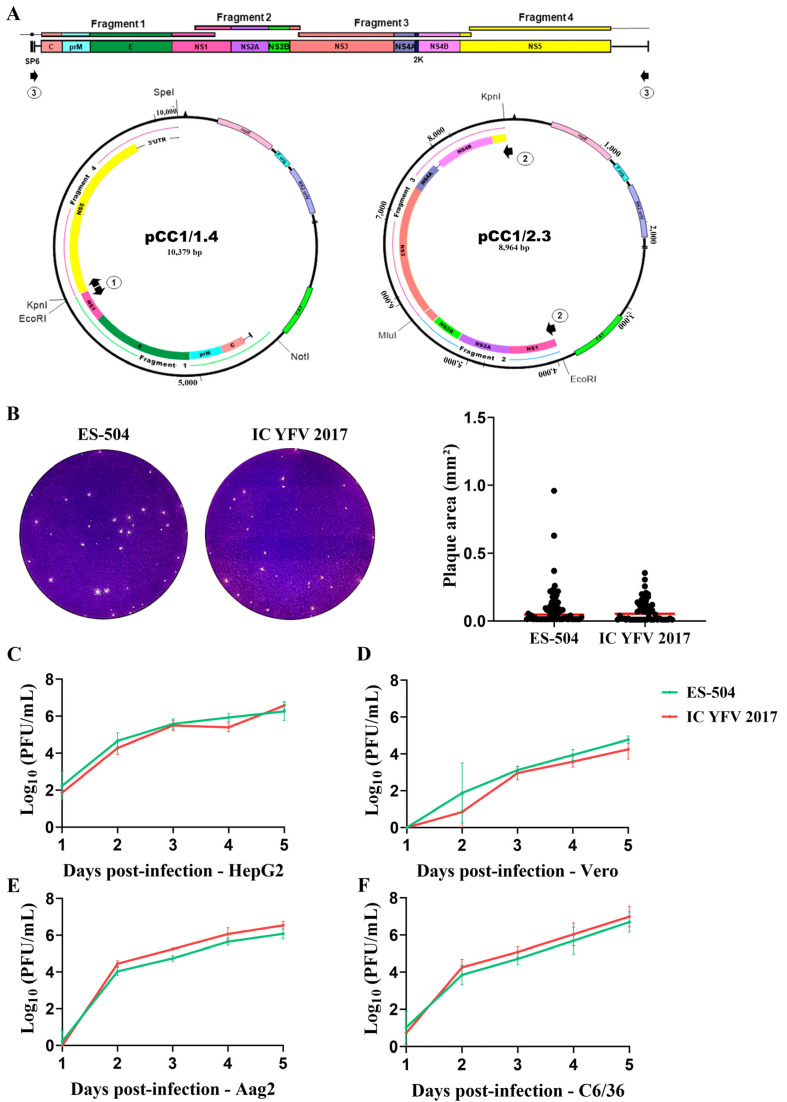
Recovery and assessment of clone-derived YFV. (**A**) Strategy for the assembly of YFV genome. The genome was divided into 4 fragments and reassembled into two main plasmids bearing the extremities (pCC1/1.4) and the central part (pCC1/2.3) of the viral cDNA. Black arrows 1 to 3 represent the primer pairs used in the amplification rounds before in vitro transcription of the template cDNA as described in Section 2.2 and Supplementary Table S1. Arrows numbered 1 represent the primer pair used to amplify the entire plasmid pCC1/1.4, arrows numbered 2, the primer pair employed in the amplification of the central part of the genome (fragment 2.3), and arrows numbered 3, are the primers used to amplify the complete viral cDNA. (**B**) Plaque morphology of parental YFV ES-504 and clone-derived virus YFV_2017. Plaque areas were measured in ImageJ software, and the results were plotted and compared in GraphPad Prism 8 with the Mann–Whitney test. (**C**–**F**) Growth curves in different cell lines: HepG2 (**C**), Vero (**D**), Aag2 (**E**), and C6/36 (**F**). The viral titers were transformed in log_10_ and plotted in GraphPad Prism 8. Statistical analyses were applied to each time point using the unpaired *t*-test to compare viral titers of YFV ES-504 and YFV_2017.

We amplified the ES-504 cDNA to obtain four fragments covering the entire genome and presenting overlapping regions (Figure 2A). The employed primer sets are listed in Supplementary Table S1. The amplification primers of fragments 2, 3, and 4 were used to insert silent mutations to create restriction sites Mlu I, Kpn I, and Spe I, allowing directional cloning in plasmid vectors. At the 5′end of fragment 1, we fused the transcription promoter sequence of bacteriophage SP6 for the following in vitro transcription step, adding a Not I restriction site. The natural EcoRI restriction site determines the 3′end of fragment 1 and the 5′ ends of fragment 2.

The plasmid vectors used to clone all four fragments were derived from pBR322 and pCC1-4K. Initially, we tried to clone the fragments in pBR322, a low-copy vector modified by the insertion of a customized multiple cloning site (MCS) carrying the selected restriction sites. Fragments 1 and 3 were successfully cloned and multiplied in *Escherichia coli* strain Sure. On the other hand, fragments 2 and 4, containing the portion of the genome that expresses active cryptic promoter sequences and the 3′end of the genome, respectively, were unstable in this vector. Thus, we used the single-copy vector pCC1-4K (GenScript) to clone these two fragments. The plasmid constructs were successfully propagated in *E. coli* strain Epi300 (Transformax kit, Epicentre). This vector was also modified to carry a customized MCS. The sequences of the MCS of both plasmid vectors are listed in Supplementary Table S1. In the end, we obtained the following plasmids: pBR322/1, pBR322/3, pCC1/2, and pCC1/4.

The genome assembly strategy adopted in this work is based on the two-plasmid system, where one plasmid bears the genome ends and another plasmid, the central part of the genome’s cDNA. Because of fragments 2 and 4’s instability, we used the pCC1-4K to vector the designed genome portions. For this, we excised fragment 1 with Not I and EcoRI (Promega) restriction enzymes digestion and ligated in plasmid pCC1/4 digested with the same restriction sites, using T4 DNA Ligase (New England Biolabs). Similarly, the plasmids pBR322/3 and pCC1/2 were digested with Mlu I and Kpn I (Promega) to excise fragment 3 and clone it into the plasmid pCC1/2. Both plasmids successfully propagated in E. coli Epi300, recovering pCC1/1.4 and pCC1/2.3 (Figure 2A).

The genome template cDNA was assembled by in vitro ligation of fragment 2.3 and plasmid pCC1/1.4. First, fragment 2.3 and the entire plasmid pCC1/1.4 were amplified using primer sets 1 and 2, listed in Supplementary Table S1. Both amplicons were digested with EcoRI and Kpn I (Promega) and ligated with T4 DNA Ligase (New England Biolabs). The product of ligation was amplified with primer set 3 (Supplementary Table S1) and subsequently in vitro transcribed with SP6 RNA Polymerase, using mMESSAGE mMACHINE SP6 Transcription kit (Ambion).

Finally, the transcribed RNA was electroporated in C6/36 cells in 0.2 mm cuvettes with 2 pulses of 400 volts, a capacitance of 25 µf, and a resistance of 800 Ω. The transfected cells were recovered in Leibovitz’s L-15 medium (Gibco) supplemented with 5% fetal bovine serum (FBS, Cutilab) and were incubated at 28 °C for 7 days. On day 7 post-transfection, cell supernatant was collected and used to detect viral genome by qRT-PCR using TaqMan Fast Virus 1-Step Master Mix as described elsewhere [44].

The viral suspension stocks used in this study were obtained from the infection of C6/36 cells, previously seeded at 80,000 cells/cm^2^ in T-75 flasks, with 3 mL of cell supernatant harvested after transfection. After 5 days of incubation at 28 °C, the supernatant was collected, centrifuged at 4 °C, 700× *g* for 10 min, filtered with 0.22 µm syringe filter, and stored at −80 °C. RNA of viral stocks was extracted using the QIAmp Mini Viral RNA kit (Qiagen) and sequenced as described elsewhere [6].

To obtain the clone-derived YFV bearing modifications at the NS5 MTase domain, we inserted 3 mutations, one by one, in plasmid pCC1/1.4 using QuikChange II XL Site-Directed Mutagenesis Kit (Agilent). The mutagenesis primers are listed in Supplementary Table S1. The mutations exchanged codons AGG (7944 to 7946), ATA (8056 to 8058), and AGC (8161 to 8163) to AAG, GTG, and GGC, generating the amino acid changes NS5 R101K, I138V, and S173G, respectively. The mutated plasmid pCC1/1.4/MTase was transformed in *E. coli* Epi300 and substituted pCC1/1.4 in the infectious clone methodology described above to recover the mutated YFV, referred to as YFV_MTase_2010. All cDNA cloning and viral recovery steps were confirmed by the whole sequencing using primer sets and the methodology described by Gómez et al. [6].

### 2.3. Plaque Phenotype Assays

The plaque phenotype assay was conducted as described elsewhere [45]. In summary, Vero cells seeded at 40,000 cells/cm^2^ were infected with 10 PFU, 20 PFU, and 40 PFU of each virus. After infection, cells were overlaid with 0.5% agarose prepared in Earle’s 199 medium (Gibco, Waltham, MA, USA) supplemented with 5% fetal bovine serum (FBS; Cutilab, Campinas, SP, Brazil), 0.25% sodium bicarbonate (Sigma-Aldrich, Saint Louis, MO, USA), and 40 mg/mL gentamicin (Gibco, Waltham, MA, USA) and further incubated at 37 °C and 5% CO_2_ for 7 days. The cells were fixed in 10.0% formaldehyde and stained with 0.4% violet crystal. Images of the plates were analyzed using ImageJ software version 1.51 to measure plaque areas. The results were plotted and statistically analyzed using GraphPad Prism software 8. Statistical tests employed were Kruskal–Wallis with Dunn’s multiple comparison test.

### 2.4. Viral Growth Kinetics in Different Cell Lines

In our study, we used Vero, HepG2, C6/36, and Aag2 cells. Vero cells were cultivated at a cell density of 40,000 cells/cm^2^ in supplemented Earle’s medium. HepG2 was seeded at 60,000 cells/cm^2^ in DMEM (Gibco, Waltham, MA, USA) supplemented with 10% FBS and 0.1% nonessential amino acids (NEAA; Gibco, Waltham, MA, USA). Both cell lines were maintained at 37 °C, with 5% CO_2_ and a wet atmosphere. C6/36 and Aag2 cells were cultivated at 80,000 cells/cm^2^ in Leibovitz’s L-15 Medium, and Schneider’s Insect Medium (Gibco, Waltham, MA, USA) supplemented with 5% and 10% FBS, respectively, and were incubated at 28 °C.

Cells were seeded in T-25 flasks with predetermined densities 24 h before infection. Viruses were diluted with an appropriate medium to infect cells at MOI 0.02 in a final volume of 0.5 mL. The cell medium was discarded, and the inocula were adsorbed in the cell monolayer for 1 h. After that, the viral suspension was discarded, the appropriate cell medium was added to the monolayer, and the cells were incubated for 5 days. An aliquot of cell supernatant was collected every 24 h and titrated by plaque assay [45]. The viral titers were transformed in log_10_ and plotted in a dispersion graph for analysis. The statistical analyses were conducted in GraphPad Prism 8 software.

### 2.5. Viral Infection in the Presence of Type I Interferon

The viral sensitivity by IFN-I was carried out as previously described [45]. Briefly, Vero cells were seeded in a 24-well plate at a density of 50,000 cells/cm^2^ 24 h before IFN treatment. Cells were pre-treated with IFN-α (PBL Assay Science, Piscataway, NJ, USA) or IFN-β (R&D Systems, Minneapolis, MN, USA) concentrations of 1000, 100, 50, and 10 UI/mL for 6 h before infection. Viral adsorption was carried out for 1 h with an MOI of 0.5. Finally, cells were overlayed with a culture medium containing the same concentrations used in pre-treatment and incubated for 48 h. The infective virus was quantified by plaque assay titration. IC_50_ values were obtained after nonlinear curve fitting available in GraphPad Prism 8 software ([Inhibitor] vs. normalized response—Variable slope).

### 2.6. Viral Inhibition by Sinefungin

Before the viral inhibition assays, HepG2 cells were tested for cell viability in concentrations ranging from 5 to 0.00032 mM of Sinefungin (Sigma-Aldrich, Saint Louis, MO, USA). Therefore, cells were seeded in 96-well plates at a density of 10,000 cells/well 24 h before treatment with Sinefungin. Cell medium was discarded, and 90 µL of supplemented DMEM medium with 5-fold serial diluted concentrations of Sinefungin was added to the cells. Treatment was carried through for 42 h in an appropriate incubator, after which 10 µL of PrestoBlue Reagent (Thermo Fisher Scientific, Waltham, MA, USA) was added to each well with treated and untreated cells. The cells were incubated for 30 min before absorbance values from each well were obtained at 570 nm normalized at 600 nm wavelength using VersaMAX Microplate Reader (Molecular Devices, San José, CA, USA).

One day before the viral infection, HepG2 cells were seeded in 24-well plates at a density of 60,000 cells/well. The viruses YFV_2017 and YFV_MTase_2010 were diluted to infect cells at MOI 0.1. Cell supernatant was discarded, 50 µL of the viral suspensions were added to the monolayer and 50 µL of supplemented DMEM medium with 4.0, 3.0, 1.0, 0.6, 0.12, 0.24, and 0.0 mM of Sinefungin. Adsorption occurred during 1 h of incubation at 37 °C, with a wet atmosphere and 5% CO_2_ with agitation every 15 min. After this step, 400 µL of supplemented DMEM medium with the corresponding concentrations of Sinefungin were added to each well. Cell supernatants were harvested after 42 h of incubation at 37 °C, with a wet atmosphere and 5% CO_2_. Viral yields were quantified by plaque assay titration in Vero cells. Viral titers were transformed in log_10_, normalized by the titer of untreated infected cells, and used to calculate IC_50_ values in GraphPad Prism 8 software with the nonlinear regression fitting algorithm of [Inhibitor] vs. normalized response—Variable slope. This experiment was performed in 4 replicates, and the IC_50_ values obtained in every replicate were analyzed by Unpaired *t*-test also in GraphPad Prism 8.

### 2.7. Neurovirulence in BALB/c Mice

BALB/c mice were purchased from CEMIB (Centro Multidisciplinar para Investigação Biológica na Área da Ciência em Animais de Laboratório) of the State University of Campinas, São Paulo (UNICAMP). Animal experimentation was carried out in accordance with the Guide of the National Council for Control of Animal Experimentation (CONCEA). The protocols employed were approved by the Committee on the Ethics of Animal Experimentation of Oswaldo Cruz Institute (CEUA-IOC; Permit: L-034/2019). Groups of 4 young adult BALB/c mice (6 weeks old) were inoculated intracerebrally with 10^3^ PFU in a final volume of 30 µL of each virus. Mock-infected mice were inoculated with the diluent medium in which the viral inocula was prepared (Earle’s 199 medium supplemented with 25 mM HEPES and 0.025% sodium bicarbonate). The mice were anesthetized with a Ketamine/Xylazine cocktail at 100 mg/kg and 10 mg/kg, respectively, administered intraperitoneally. When no righting reflexes were detected, the mice were inoculated. The animals were monitored daily for 16 days with the evaluation of clinical signs of disease and weight measurement. Clinical scores were established to determine the humane endpoint for the euthanasia of mice, as previously described [45]. Evaluated clinical signs included the percentage of body weight loss, ruffled fur, hunched posture, low mobility, paralysis of posterior members, aggressiveness, and respiratory disorders. Euthanasia was performed with intraperitoneal administration of three times the previous dose of the Ketamine/Xylazine cocktail, followed by cervical dislocation. This experimentation was reproduced in quadruplicate, totalizing 16 animals infected with each viral sample. Average survival time (AST), percentage of mortality, clinical scores, and body weight loss were calculated and analyzed in GraphPad Prism 8 software. Statistical analysis of Kaplan–Meier survival curves was performed by log-rank test (Mantel–Cox).

### 2.8. Expression and Purification of YFV NS5 MTase Domain

For the expression and functional studies, the sequence corresponding to the methyltransferase domain of YFV NS5 (aa 1-270) was amplified using specific primers with Phusion DNA Polymerase and reagents (Thermo Fisher Scientific). The amplicon was obtained from ES-504 isolate (GenBank: KY885000) and then cloned into a pETM-11/LIC plasmid vector with an N-terminal His6 tag. The vector plasmid was modified with the deletion of the connector sequence and the TEV cleavage site between the His6 tag and the MTase domain by PCR with Phusion DNA Polymerase. The variant carrying the reverted amino acids of the molecular signature (R101K, I138V e S173G) was obtained through site-directed mutagenesis using QuikChange II XL Site-Directed Mutagenesis Kit (Agilent). Amplification, mutagenesis, and deletion primers are listed in Supplementary Table S1.

Both MTase variants were produced in a T7 Express Competent *Escherichia coli* C2566 (New England BioLabs). Bacteria were grown in Terrific Broth at 37 °C until the optical density at 600 nm (OD_600_) reached 0.6. Protein expression was then induced by 1 mM IPTG (isopropyl-β-d-thiogalactopyranoside) and 2% ethanol at 17 °C overnight. Then, bacteria were harvested by centrifugation at 10,000× *g* a 4 °C for 10 min. The bacterial pellets from 2-L bacterial culture were resuspended in lysis buffer (50 mM Tris-HCl [pH 8], 300 mM NaCl, 10% glycerol, 1 mM β-mercaptoethanol, 10 μg/mL DNase I, 1 mM PMSF, 0.25 mg/mL lysozyme, and 1 mL/10 mL of BugBuster Protein Extraction Reagent). After 30 min of incubation at 4 °C, the cells were sonicated for 2 min every 30 s, with an amplitude of 45%, and clarified by centrifugation. Then we proceeded to immobilized metal affinity chromatography (IMAC) purification on a 5-mL His prep column (GE Healthcare), with elution in 50 mM Tris-HCl, 300 mM NaCl, and 250 mM imidazole (pH 8.0). The eluted protein was then dialyzed and stored in a mixture of 10 mM HEPES-NaOH, 500 mM NaCl, glycerol 5%, and 1 mM dithiothreitol (DTT) [pH 7.5]. Proteins were finally concentrated using centrifugal filters of 30 kDa and stored at −20 °C after adding glycerol to a final concentration of 40%.

### 2.9. Thermal Shift Assay

The purified MTase proteins were diluted to the concentration of 0.5 mg/mL in 25 µL of protein storage buffer. SYPRO Orange Fluorescent Dye (Bio-Rad) was diluted 500 times in buffer. Then the reaction was set up with 21.5 µL of diluted protein and 3.5 µL of diluted dye. The thermocycler (Bio-Rad TFX8016) ran a reaction with the following settings: an initial 2 min hold at 25 °C, ramping up in increments of 1 °C to a final temperature of 95 °C, with a 2 min hold.

### 2.10. Radioactive Methyltransferase Assay

The enzymatic assays were carried out in 40 mM Tris-HCl (pH 8.0), 1 mM DTT, 0.1 µM [3H]SAM (PerkinElmer), and 1.9 of SAM in the presence of 0.7 µM synthetic RNA with m7GpppAC5. The purified MTase domains were added to a final concentration of 0.1 µM. To determine the enzymatic parameters, SAM and RNA concentrations varied from 0.0 to 3.0 µM. The MTase competition assays occurred in similar conditions as described above, except for the final concentration of YFV MTase in the reactions of 0.5 µM.

The reaction mixtures were incubated at 30 °C and stopped after 30 min by a 10-fold dilution of the reaction mixture in ice-cold Milli-Q water supplemented with SAH 1 µM. Samples were then transferred to DEAE cellulose filters (PerkinElmer) using Filtermat Harvester (Packard Instruments). Before a final drying step, the unincorporated [3H]SAM was washed from the DEAE filters with 10 mM ammonium formate (pH 8.0), with H_2_O, and with absolute ethanol. At last, the filters were placed into plastic bags with BetaplateScint (Wallac) scintillation fluid and sealed. The transferred [3H]methyl groups onto RNA substrates were quantified in counts per minute using a Wallac 1450 MicroBetaTriLux liquid scintillation counter. The IC_50_ values of SAH, sinefungin and cap analogs were determined with GraphPad Prism software 8 using the log (inhibitor) versus response variable slope equation.

## 3. Results

### 3.1. Structural Analysis of the Molecular Signature in the NS5 MTase Domain

A previous study on the circulating YFV 2016−2019 first explored the molecular signature in computational tridimensional structure analyses of NS3 and NS5 viral proteins [6]. Here, we reproduced the comparative modeling methodology, focusing exclusively on the MTase domain, to review the structural localization and potential influence of the three residues characteristic of YFV 2016−2019, which caused the outbreak in Southeastern Brazil. Thus, we modeled the MTase domain of YFV 2016−2017 NS5 protein by homology, using the crystallographic structure of YFV 17D MTase (PDB: 3EVB), sharing 94.32% of identity and 100% coverage, as a template.

The YFV 2016−2019 molecular signature in the MTase consists of residues R, I, and S in positions 101, 138, and 173, respectively. These residues are adjacent with distances between Cα from 101 to 138 and 138 to 173 of about 10Å. These amino acids are not directly involved in binding or catalytic interactions; however, they are closer to the methyl donor (SAM) binding site (Figure 1).

We also utilized the PyMOL software to change the three amino acid alterations, reverting these residues to the corresponding previously circulating YFV 2000−2010, here named as MTase 2010. The substitutions were Arg (R) to Lys (K) in position 101, Ile (I) to Val (V) in position 138, and Ser (S) to Gly (G) in position 173. All three-residue changes are conservative and have little impact on the 3D structure. Notably, residues with longer lateral side chains substituted the residues of MTase 2010. For instance, the R residue in 101 (MTase 2017) has an additional amino group in the side chain than the K residue (MTase 2010). Additionally, the I residue (MTase 2017) in 138 has an additional methyl group than the V amino acid side chain (MTase 2010). The longer side chains might potentially reduce the distance between amino acids 101 and 138 and slightly disturb the SAM binding site (Figure 1).

### 3.2. Recovery of Clone-Derived YFV

We obtained infective viruses using the infectious clone methodology to determine whether the YFV 2016−2019 molecular signature in the MTase domain of NS5 protein influences viral replication. Here, we adapted the methodology described previously for the Zika virus Rio-U1 infectious clone [43].

The genome of the YFV ES-504 isolate was amplified in four different fragments by RT-PCR. All fragments were engineered to carry unique restriction sites at their ends to direct cloning and subsequent genome assembly. The first fragment was also fused to the bacteriophage SP6 promoter sequence for in vitro transcription. Each fragment was cloned into plasmid vectors, after which two main plasmids were constructed, carrying two fused fragments each: pCC1/1.4 and pCC1/2.3. These plasmids were used to obtain the YFV ES-504 clone-derived virus, YFV_2017, upon restriction treatment followed by DNA ligation, PCR amplification of template cDNA, in vitro transcription, and transfection in C6/36 cells (Figure 2A). Viral recovery was confirmed by qRT-PCR detection with 1.28 × 10^8^ genomic RNA copies/mL.

The validation of YFV_2017 as a comparable infective virus to its parental strain ES-504 was performed in two steps. First, we compared the plaque morphology of both clone-derived and parental viruses. Although ES-504 plaque showed more heterogeneity in area, there was no significant difference compared to YFV_2017 (*p* = 0.213) (Figure 2B). Next, we evaluated the viral growth kinetics in four cell lines: two derived from mammal hosts (Vero and HepG2) and two derived from mosquitoes (Aag2 and C6/36). The viral replication in all cell lines was similar without statistical significance (Figure 2C–F). Therefore, we concluded that the infectious clone-derived virus YFV_2017 is a suitable model for studying mutations’ effect in YFV.

### 3.3. Cell Infection Studies with the Mutant YFV/MTase 2010

To study the effects of the YFV 2016−2019 molecular signature in the NS5 MTase domain, we inserted mutations in the pCC1 + 1.4 plasmid, changing the three amino acid residues that compose the signature in this domain: R101K, I138V, and S173G. This mutated plasmid was used to assemble the viral cDNA further and recover the YFV variant MTase_2010. Viral recovery was confirmed by qRT-PCR with 2.27 × 108 genomic RNA copies/mL. The effects of this set of mutations on viral fitness were next investigated in cell cultures and BALB/c mice.

**Figure 3:**
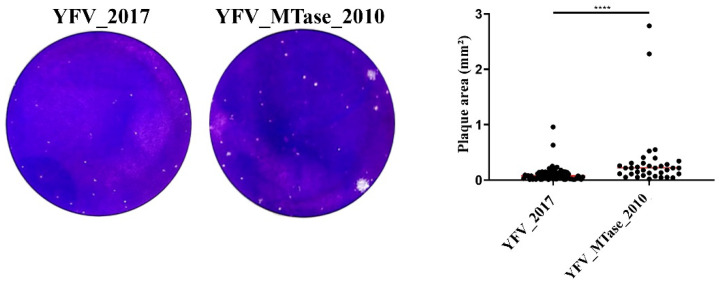
YFV_2017 and YFV_MTase_2010 plaque phenotype determination by area measurements. Red lines represent the area median value. Plaque areas were measured in ImageJ software, plotted in GraphPad Prism 8, and compared by Mann–Whitney test. **** represents the statistical significance of *p* < 0.0001.

First, we compared the plaque morphology of both the YFV_2017 and YFV_MTase_2010 viruses (Figure 3). Although both viruses presented, in general, tiny plaques, we observed larger plaque sizes in YFV_MTase_2010 than in its parental clone-derived virus YFV_2017, with statistically significant differences (*p* < 0.0001).

**Figure 4:**
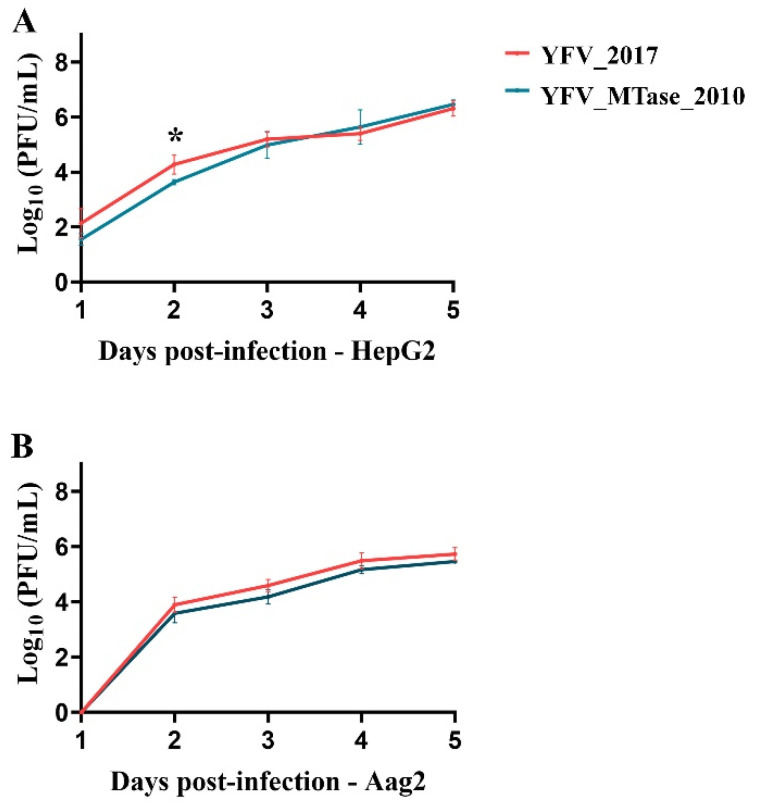
Viral yields of YFV_2017 and the YFV_MTase_2010 viruses in infected (**A**) HepG2 cells and (**B**) Aag2 cells. The viral titers were transformed in log10 and plotted in GraphPad Prism 8. Statistical analyses were applied to each time point using the unpaired *t*-test to compare viral titers of YFV_2017 and YFV_MTase_2010. The asterisk in (**A**) represents the statistical significance with *p* = 0.0361.

We further compared the ability of the parental IC YFV 2017 and the IC YFV/MTase 2010 viruses to replicate in human (HepG2) and Aedes aegypti (Aag2) cells (Figure 4). In HepG2 cells, the IC YFV/MTase 2010 tended to replicate less than the IC YFV 2017 until 3 days post-infection (dpi). However, only at 2 dpi we detected a statistical difference between the YFV variants (*p* = 0.0361). After 4 dpi, the viral growth of IC YFV 2017 and IC YFV/MTase 2010 became almost indistinguishable. On the other hand, no statistical differences were observed in Aag2 cells during the experiment.

**Figure 5:**
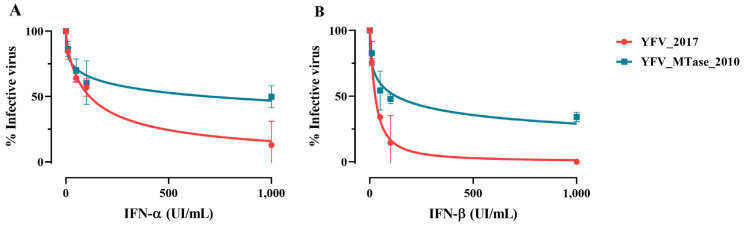
Effect of IFN-I cell treatment in viral proliferation. (**A**) The nonlinear fit of viral growth in the presence of distinct concentrations of IFN-α and (**B**) IFN-β for YFV_2017 and YFV_MTase_2010. Data were analyzed in GraphPad Prism 8 with nonlinear regression [Inhibitor] vs. normalized response—Variable slope.

Considering the ability of flaviviruses to evade innate immune response mediated by interferon (IFN) and that the NS5 protein interplays directly and indirectly with this pathway, we evaluated if the amino acid alterations could interfere in YFV sensitivity to type-I IFN. We treated Vero cells with 1000, 100, 50, and 10 UI/mL of IFN-α and IFN-β for 6 h; then, we infected those cells with YFV_2017 and YFV_MTase_2010 at MOI 0.5. After viral adsorption, cells were incubated with the same concentrations of IFN for 48 h. The viral titers at each culture condition were determined and transformed in log_10_ to fit in a nonlinear regression to calculate IC_50_ (Figure 5).

YFV_2017 was more sensitive to IFN-α and IFN-β treatments, presenting lower IC_50_ values than YFV_MTase_2010 (Figure 5). The IC_50_ displayed by YFV_2017 was 115.4 UI/mL and 26.66 UI/mL for IFN-α and IFN-β, respectively. Whereas those values for YFV_MTase_2010 were 693.6 UI/mL and 126.6 UI/mL under treatment with IFN-α and IFN-β, respectively.

**Figure 6:**
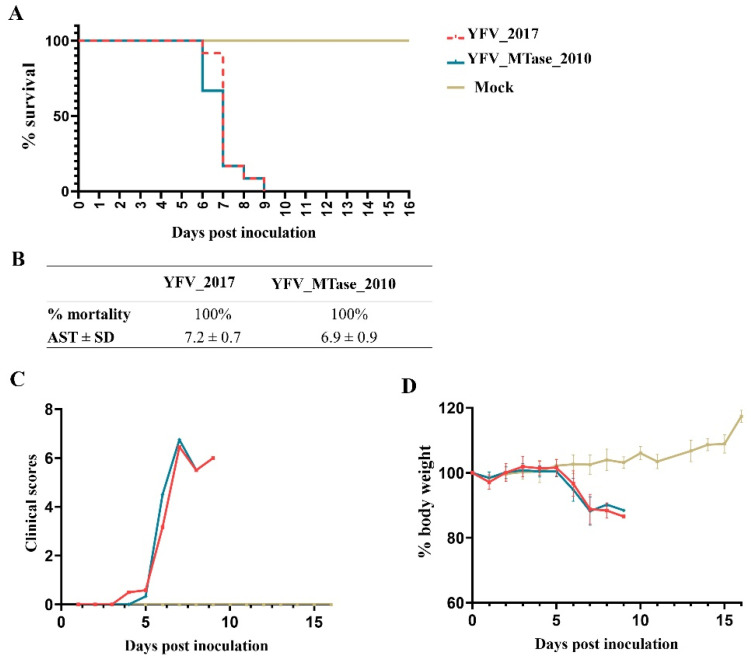
Neurovirulence in BALB/c mice after inoculation with YFV_2017 and YFV_MTase_2010. (**A**) Kaplan–Meier survival curves of animal groups inoculated with each virus and culture medium (Mock). (**B**) Table summarizing the results obtained from the survival curves. (**C**) Bodyweight measurements during the 16 days after inoculation. (**D**) Clinical scores of the animals until euthanasia.

Finally, we assessed viral fitness through a neurovirulence test in a mouse model. Young adult BALB/c mice were inoculated intracerebrally with 10^3^ PFU of each virus and monitored daily for body weight loss and clinical signs of disease. YFV_2017 and YFV_MTase_2010 provoked 100% lethality on the ninth day after inoculation. The average survival time of mice inoculated with YFV_2017 and YFV_MTase_2010 was 7.2 ± 0.7 and 6.9 ± 0.9 days, respectively. The survival curves were compared statistically by the Log-rank (Mantel-cox) test and were not significantly different (*p* = 0.4224). Likewise, the disease outcome and body weight loss were similar between the two groups (Figure 6).

### 3.4. In Vitro Inhibition by SAM Analog Sinefungin

**Figure 7:**
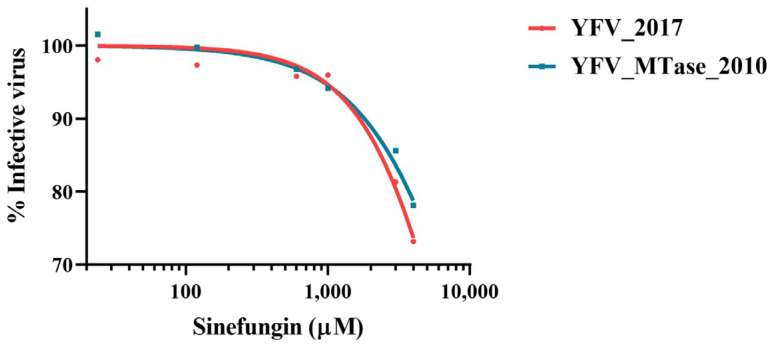
Viral replication in the presence of Sinefungin. The nonlinear fit of viral activity in the presence of the inhibitor molecule. Each point represents the result of four independent inhibition experiments. The nonlinear regression was conducted with the algorithm [Inhibitor] vs. normalized response—variable slope of GraphPad Prism 8.

To deepen the study on the influence of the YFV 2016−2019 molecular signature, we tested the inhibition of viral growth by a nucleoside S-adenosyl-L-methionine (SAM) analog, Sinefungin. HepG2 cells were infected with YFV_2017 and YFV_MTase_2010 in the presence of different concentrations of Sinefungin for 42 h, after which the cell supernatants were harvested and used for viral quantification by plaque assay. Viral titers were transformed in log_10_ and used to represent a nonlinear regression curve fit. The results show that Sinefungin inhibited the two viruses with similar levels (Figure 7). The inhibitor was not efficient against the infection of HepG2 cells with both YFV, with the lowest percentage of infection around 70%, not reaching 50% of inhibition even at the concentration of 4 mM. No apparent difference was observed between YFV_2017 and YFV_MTase_2010.

### 3.5. Protein Expression and Enzymatic Activity

**Figure 8:**
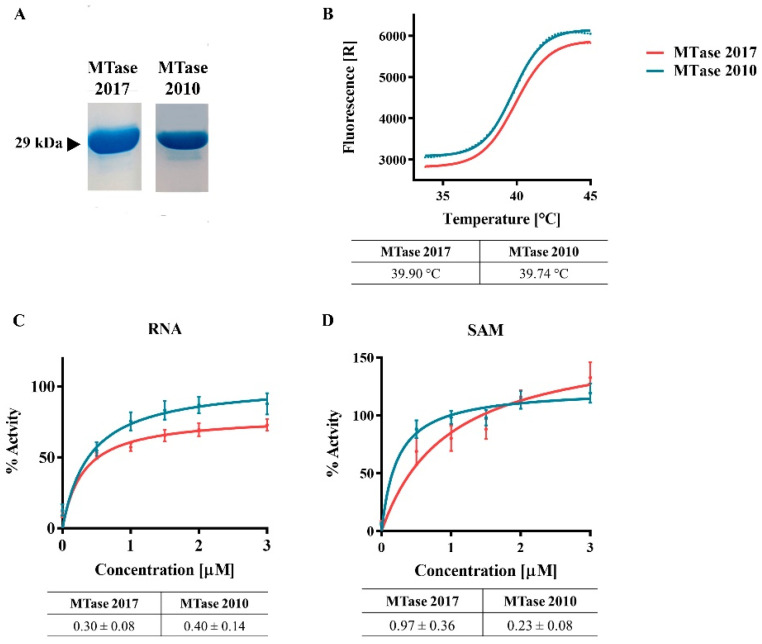
MTase expression and activity. (**A**) SDS-PAGE of purified proteins MTase 2017 and MTase 2010. (**B**) Thermal shift assay of the two MTase variants, with their respective melting temperature. (**C**,**D**) Michaelis-Menten curve fit of MTase activities and the Km values using varying concentrations of RNA or SAM (**D**).

The MTase domain of the YFV 2016−2019 NS5 and a variant carrying the three mutations R101K, I138V, and S173G were expressed in E. coli C2566 pRARE2. Both isoforms were purified by IMAC and tested to identify the differences in stability and function between MTase 2017 and MTase 2010 (Figure 8). After protein purification, we performed a thermal shift assay and determined the melting temperatures (Tm) of both MTase. We observed that the mutations did not influence protein stability, and both proteins’ Tms are about 40 °C (Figure 8B).

To assess the influence of the mutations on the MTase activity, we compared the enzymatic activity of both proteins using small synthetic capped RNA with (^m7^GpppAC_5_) for Km determination upon Michaelis-Menten curve fitting (Figure 8C,D). Whereas the Km values for the RNA substrate were similar for both proteins, the Km values for the methyl donor (SAM) were increased for MTase 2017, indicating a lower affinity for the SAM (Figure 8D).

**Figure 9:**
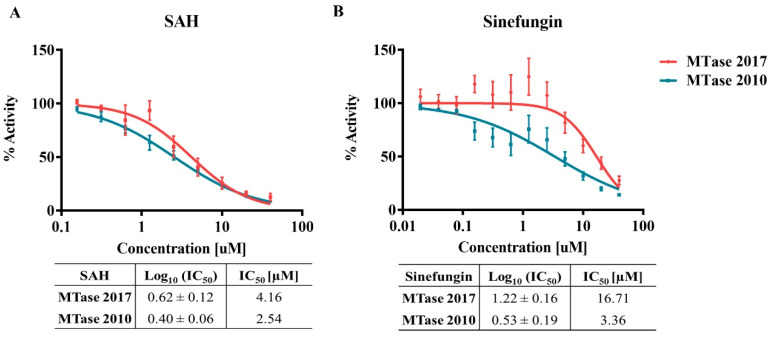
Competition enzymatic assay with the isoforms MTase 2017 and MTase 2010 in the presence of SAM analogs. (**A**) MTase inhibition by SAH and (**B**) Sinefungin. The IC50 values are in the corresponding tables below the graphs.

We further investigated the effect of the mutations by determining the inhibition of both MTase variants by S-adenosyl-L-homocysteine (SAH), the natural by-product of the methyltransferase reaction and the allosteric inhibitor Sinefungin (Figure 9). The two competitive inhibitors were shown to inhibit slightly more efficiently the MTase 2010 than the MTase 2017. Although there were no statistical differences, the divergence between inhibition of MTase 2010 and MTase 2017 was more pronounced upon treatment with Sinefungin (*p* = 0.35 for SAH, and *p* = 0.2, for Sinefungin). These results show that the mutations may interfere with the interaction between the MTase and the methyl donor, SAM, which is essential for the reaction turnover.

## 4. Discussion

The 2016−2019 YFV outbreak in southeastern Brazil is a consequence of complex factors involving ecological and biological landscapes and non-stochastic factors. The genomic characteristics of the YFV variant circulating in Southeast Brazil from 2016 to 2019 might also have played a role [46]. We determined that viral strains associated with the most severe YF epidemic in South America in the last 80 years displayed a set of nine unique amino acid polymorphisms located in highly conserved positions in nonstructural proteins [6]. Three of the residue changes occurred at the NS5 MTase domain, which is crucial to the viral replication cycle. Indeed, this domain plays key roles in RNA capping and methylation, which protect viral RNA from degradation and sensing by innate immunity. Modifications in this protein can thus interfere directly with its enzymatic activity, exposing the viral RNA to the host’s innate immune response and suppressing viral replication [15,24].

Additionally, acidic amino changes in the MTase domain of NS5 might also influence the interactions between cellular and viral proteins, modulating the host’s antiviral response. Indeed, the NS5 MTase domain has also been described to interfere with proteins, such as STAT2, in the interferon-induced signaling pathway [30,31,32,34,35,36]. Here we characterized the effects of the YFV 2016−2019 molecular signature in the MTase in the context of viral replication and virulence and its specific catalytic function.

We deciphered the effect of MTase variations initially by constructing two synthetic YFVs. The first synthetic YFV, IC YFV 2017, corresponds to the isolate ES-504, belonging to the lineage of YFV variants that caused the 2016−2019 outbreak. The synthetic virus displayed a similar phenotype to its parental wild-type isolated YFV ES-504, allowing its use as a reference to study genetic markers. The second synthetic virus consists in the backbone of the IC YFV 2017, in which the amino acid changes (R101 to K, I138 to V, and S173 to G) carried by previously circulating YFV (2000−2010) were introduced in the NS5 MTase coding sequence. The MTase 3D model suggested that these amino acids are located at the protein’s surface and are not directly involved in SAM or RNA binding or catalytic interactions. However, they are proximal to the methyl donor binding site.

Our results reveal that the effect of the three amino acid changes in the MTase domain is modest, and the viruses show similar replication and virulence, with only subtle differences. YFV_MTase_2010 exhibited a slightly larger plaque size morphology than YFV_2017. The viral growth curves in a mammal (human hepatocytes: HepG2) and mosquito (Aedes aegypti larvae: Aag2) cells were similar. In contrast, the infection of Vero cells in the presence of IFN-I led to distinguished phenotypes between YFV_MTase_2010 and YFV_2017. This indicates that the residues 101, 183 and 173 of NS5 play a role in the viral escape of innate immune response mediated by IFN-I. Indeed, the MTase domain of flaviviruses protein NS5 has been described to interact with elements of the IFN-I pathway [36,47].

The results obtained in Vero cells infected in the presence of IFN-I led us to determine whether this difference would result in a significant phenotype of the infection in a mammal model. For this purpose, we performed a neurovirulence assay, a classic method used to characterize YFV, as the vaccine YFV 17D is highly neurovirulent [48]. This test reveals insights about YFV fitness in complementarity to experiments of virulence in cellular model studies. We inoculated intracerebrally young adult BALB/c mice to compare the neurovirulence of YFV_2017 with YFV_MTase_2010. However, our results showed no significant difference in neurovirulence, suggesting that the mutations did not influence YFV fitness in vivo.

Finally, the HepG2-cell infection with the two variant viruses in the presence of Sinefungin, a SAM analog, did not present any substantial difference. On the other hand, the biochemical approach highlighted one potential role of the three mutations on the function of MTase. We observed that MTase 2010 displays a more pronounced affinity for SAM. This result suggests that these residues might be involved in SAM/SAH turnover, which is consistent with the localization of the mutated residues (Figure 1). Indeed, the three residues might function as an allosteric pocket, which means that the conformational changes in the surface of MTase could provoke a change in the binding pocket’s dynamics [49]. We further confirmed the greater affinity of the MTase 2010 with the methyl donor in a competition assay with SAH and Sinefungin, antagonists of SAM. In both assays, the SAM analogs showed more potent inhibition of the MTase 2010, but the difference in inhibitory effect is more pronounced for Sinefungin. These results suggest that modifying the affinity for the SAM molecule might play a role in both N7- and 2′O-methylations, as the NS5 MTase contains a unique SAM binding mode for both reactions. Two models for the cap methylation of flaviviruses have been proposed. One model postulates that a single MTase molecule performs both methylations, requiring the dissociation of the first by-product after N7-methylation and the re-association of another SAM molecule to perform the 2′O-methylation [16,23]. The other model implies that two molecules of MTase are required to complete both cap methylations of viral RNA [50]. A higher affinity to the SAM molecule might slow down the methyl transfer reaction in both situations. Therefore, the efficiency of the MTase 2017 is slightly affected.

In summary, our results show that the YFV 2016−2019 strains’ molecular markers in the MTase domain do not affect YFV virulence. However, it caused small changes in the SAM binding properties, but not enough to be detected using the complete viral particles YFV_2017 and YFV_MTase_2010 in the cell infection model. These residues might modulate the antiviral response against YFV mediated by type I interferon, which needs further investigation. This study contributed to the molecular basis of the evolution of genotype South America I YFV in Brazil by characterizing the effects of amino acid mutations that naturally occurred in the YFV circulating since the outbreak of 2016−2019. Our results point to further studies on mapping the residues in the NS5 protein that interact with the elements of the IFN signaling pathways.

## Data Availability

Not applicable.

## Supplementary Materials

The following supporting information can be downloaded at: https://www.mdpi.com/article/10.3390/v15010191/s1, Table S1: Oligonucleotide sequences for the infectious clone methodology and site-directed mutagenesis.
