# Supplementary material for: Amino Acid Polymorphisms on the Brazilian Strain of Yellow Fever Virus Methyltransferase Are Related to the Host’s Immune Evasion Mediated by Type I Interferon
Source: Viruses. 2023 Jan 10;15(1):191. doi: 10.3390/v15010191 (PMC9863089; doi:10.3390/v15010191)
Supplement: Supplementary file 1 [file viruses-15-00191-s001.zip › viruses-2072658-supplementary.pdf]

TableS 1 - Oligonucleotide sequences for the infectious clone methodology and site-directed mutagenesis

	Forward Primer		Reverse Primer		Purpose
	**ID**	**Sequence (5' --> 3')**	**ID**	**Sequence (5' --> 3')**	
Cloning steps	1(+)	CGCCTGGTATCTTTATAGTCCTGTCGG GTTTCGC	1(-)	CCAAATGTGTTTATTGCCTAGCAA CTCGATTTGCAGACC	Merge the SP6 transcription promoter to fragment 1
	ICYFSP6	ATTTAGGTGACACTATAGAGTAAATC CTGTGTGCTAATTGAGGTGCATTGG	2.1(-)	TTGACCTCGGCATGAACATGTCAC TCTCTTCAA	Obtain fragment 1
	3(+)	GAATGGAAGCTTCATCATCGATGGGA AGTCCAGGAAA	3(-)	GAGAAAGGCCCCACGCGTGACGTG C	Obtain fragment 2
	4(+)	GCACGTCACGCGTGGGGCCTTTCTC	4(-)	TCTCAATTTTGCGGTACCTCTCGAG ACGGCC	Obtain fragment 3
	5(+)	GGCCGTCTCGAGAGGTACCGCAAAAT TGAGA	5(-)	GAGCCAGACGGACTAGTGGTTTTG TGTTTGTCATCC	Obtain fragment 4
	MCS2(+)	GGCCGCCATATGATCGATGCGCGCGA ATTCACGCGTGACGTCGGTACCGGCG CCGTGCACACTAGTATGCA	MCS2(-)	TACTAGTGTGCACGGCGCCGGTAC CGACGTCACGCGTGAATTCGCGCG CATCGATCATATGGC	MCS of pCC1-4K
	MCS3(+)	GGCCGCCATATGATCGATGCGCGCGA ATTCACGCGTGACGTCGGTACCGGCG CCGTGCACACTAGTC	MCS3(-)	TCGAGACTAGTGTGCACGGCGCCG GTACCGACGTCACGCGTGAATTCG CGCGCATCGATCATATGGC	MCS of pBR322
Template cDNA regeneration	Mlu(+)	TTCACGCGTGACGTCGGTACCGCAAA ATTG	Mlu(-)	ACGCGTGAATTCCACACTCTGTTT GAGAAC	Primer set 1 - Amplification of the entire pCC1+1.4 plasmid
	2.3(+)	CGCGCGAATTCCTTCCAGATAGAAGA GTTT	2.3(-)	TTTTCCCAGTCACGACGTTGTAAA ACGACG	Primer set 2 - Amplification of fragment 2.3
	ICYFSP6	ATTTAGGTGACACTATAGAGTAAATC CTGTGTGCTAATTGAGGTGCATTGG	YFmelt65Rnew	AGTGGTTTTGTGTTTTTCGTCCAA AGGTCTGCTTATTCTTGAGC	Primer set 3 - Final template cDNA amplification
Site-directed mutagenesis	NS5_R101K(+)	GAGAAGTGAGTGGGGTCAAGGGATT CACCCTT	NS5_R101K(-)	AAGGGTGAATCCCTTGACCCCACT CACTTCTC	Mutagenesis in 101
	NS5_I138V(+)	CCATCGCCTTGAGCCGGTAAAGTGTG ATACCCT	NS5_I138V(-)	AGGGTATCACACTTTACCGGCTCA AGGCGATGG	Mutagenesis in 138
	NS5_S173G(+)	CTGTTGAGAAATGGTTGGGCTGTGGT GTTGAAAGC	NS5_S173G(-)	GCTTTCAACACCACAGCCCAACCA TTTCTCAACAG	Mutagenesis in 173
